# Dynamics of Gill Responses to a Natural Infection with *Neoparamoeba perurans* in Farmed Tasmanian Atlantic Salmon

**DOI:** 10.3390/ani14162356

**Published:** 2024-08-15

**Authors:** Max Charles Vallarino, Sarah L. Dagen, Eoin Costelloe, Shalom Inioluwa Oyenekan, John Tinsley, Victoria Valdenegro, Elżbieta Król, Patricia Noguera, Samuel A. M. Martin

**Affiliations:** 1 Scottish Fish Immunology Research Centre, School of Biological Sciences, University of Aberdeen, Aberdeen AB24 2TZ, Scotland, UK; m.charlesvallarino.22@abdn.ac.uk (M.C.V.); sarahlouise.dagen@gmail.com (S.L.D.); eoincostelloe@hotmail.com (E.C.); shalomoyenekan@gmail.com (S.I.O.); e.krol@abdn.ac.uk (E.K.); 2 BioMar Ltd, Grangemouth FK3 8UL, Scotland, UK; jtinsley@biomar.com; 3 BioMar Australia, Wesley Vale, TAS 7307, Australia; vicva@biomar.com; 4 Aquaculture and Marine Environment, Marine Scotland Science, Aberdeen AB11 9DB, Scotland, UK

**Keywords:** amoebic gill disease (AGD), biomarkers of gill health, complex gill disease (CGD), gene expression, gill histopathology, gill scores, angiogenin-1 (*ANG1*), C1q tumour necrosis factor-related protein 3-like (*C1QTNF3*)

## Abstract

**Simple Summary:**

This study focuses on how Tasmanian sea-farmed Atlantic salmon respond to a natural infection with a parasite that causes amoebic gill disease (AGD), a major health issue for salmon globally. The research aimed to understand the progression of the disease by examining the salmon’s gills at different stages of the outbreak and evaluating their response to freshwater treatment. The study employed both macroscopic and microscopic examinations of gills, as well as molecular tools to measure the abundance of amoebae and the expression of specific genes that indicate inflammation. Key findings showed a strong correlation between different measures of gill health and significant differences between distinct stages of AGD. After treatment, a varied response among the fish was observed, indicating that management strategies need to account for individual genetic, environmental, and health factors. The expression patterns of some inflammation-related genes highlight their potential as biomarkers for early detection of gill damage in salmon aquaculture worldwide. The results of this research are important as they can help develop better ways to detect and manage this disease early, potentially reducing losses in salmon farming and ensuring the sustainability of this vital food resource.

**Abstract:**

Gill health has become a significant global challenge for Atlantic salmon (*Salmo salar*) aquaculture, particularly during the marine phase of farming. The increasing prevalence of gill pathologies has been linked to rising seawater temperatures, underscoring the need to evaluate existing tools for monitoring gill health and to develop novel approaches for early detection. In this study, we investigated the gill responses of commercially farmed Atlantic salmon to natural infection with *Neoparamoeba perurans* during an outbreak of amoebic gill disease (AGD) in Tasmania. Our focus spanned the low AGD prevalence, high AGD prevalence, and post-freshwater treatment stages of the outbreak. Evaluations of gill tissue included assessments of the gross AGD score, histopathological score, abundance of *N. perurans* (measured by *18S rRNA* gene expression), and expression levels of inflammation-related transcripts. We demonstrated a strong correlation between different measures of AGD-related gill pathology and significant differences between distinct stages of the *N. perurans* outbreak. Post-treatment, fish exhibited considerable variability in their responses to the freshwater bath, highlighting the necessity for personalized management strategies that consider genetic, environmental, and health status factors. The expression patterns of angiogenin-1 (*ANG1*) and complement C1q tumour necrosis factor-related protein 3-like (*C1QTNF3*) emphasize their potential as biomarkers for early detection of gill damage in salmon aquaculture worldwide.

## 1. Introduction

Atlantic salmon (*Salmo salar*), extensively farmed in temperate regions worldwide, faces cultivation limits due to extreme summer and winter temperatures. The industry’s growth is further constrained by infectious and non-infectious diseases, with gill health being a significant concern. This is primarily due to infectious agents such as *Neoparamoeba perurans*, which causes amoebic gill disease (AGD) [1,2], and various agents contributing to complex gill disease (CGD) [3,4]. The increasing prevalence of gill pathologies has been linked to rising seawater temperatures associated with climate change [5,6,7].

The gill of teleost fish, including Atlantic salmon, is vital for osmoregulation, gas exchange, excretion of nitrogenous waste, and immune function [8,9]. Its extensive surface area maintains constant contact with the aquatic environment. Each gill arch consists of filaments branching into highly vascularized lamellae [10]. Gill-associated lymphoid tissues (GIALT) at the filament bases play crucial immune roles [11,12,13], including pathogen and parasite prevention [14]. When compromised, the gill undergoes non-specific pathologies such as inflammation, lamellar fusion, and epithelial hyperplasia, leading to dysfunction and reduced performance [4,15,16,17].

AGD, first identified in the 1980s [1], is a major threat to the global salmon industry [18,19]. Initially reported in Tasmania [1] and the USA [20], AGD has spread to major salmon-producing regions worldwide, including Chile, Ireland, Scotland, and Norway [21,22,23,24]. Caused by the marine amoeba *N. perurans* [2], the disease triggers gill inflammation, manifested by epithelial hyperplasia, hypertrophy, lamellar fusion, and increased mucus production [24,25,26]. Clinically, AGD presents as white mucoid lesions on the gills, with affected fish exhibiting respiratory distress, cardiac dysfunction, anorexia, lethargy, and surface congregation [20,27,28]. The disease spreads rapidly in warmer months, significantly reducing salmon’s ability to withstand environmental stressors [21,29,30]. Without timely treatment, AGD can cause up to 50% mortality [1], highlighting the need for vigilant monitoring for *N. perurans* infection.

Monitoring gill health for AGD involves non-destructive, gross morphological examination of gill arches using an AGD scoring system to evaluate white mucoid lesions [31]. Some fish also undergo histopathological examination with a scoring system for gill histopathology in sea-farmed Atlantic salmon [16,32,33]. *N. perurans* presence is confirmed microscopically or via PCR assays targeting the 18S ribosomal RNA gene [34,35,36,37]. Recent gill transcriptomics studies have identified potential biomarkers for gill inflammation, useful for monitoring gill health [9]. Each technique has limitations: gross morphological scoring can be inconsistent, especially early in the disease [9]; histopathological examination is labour-intensive and time-consuming [33]; and detecting *N. perurans* does not reveal overall gill health. The biomarker panel, developed for Scottish Atlantic salmon, may not be applicable elsewhere [9]. More research is needed to compare monitoring tools for AGD and gill health, which are crucial for early detection, treatment strategies, and treatment evaluation.

Commercial treatments for AGD include freshwater or hydrogen peroxide baths, lasting 15 min to 4 h depending on the type [24,38,39], generally administered when the average gross AGD gill score exceeds 2 [31]. Freshwater baths, used in Tasmania [38,40], parts of Ireland [36], and Norway [23], involve immersing fish in freshwater for 2 to 4 h, significantly reducing amoebae and excess mucus [38,41], though limited by freshwater availability. Hydrogen peroxide baths, effective in cooler regions like Scotland, Ireland, and Norway, use 1000–1400 mg/L for 18–22 min but are unsafe above 13.5 °C or when AGD scores reach 3 [39,42]. Rising global water temperatures make freshwater bathing the predominant AGD treatment [43,44], necessitating further research.

In this study, we investigated the gill responses of commercially farmed Atlantic salmon during a natural infection with *N. perurans* amidst an AGD outbreak in Tasmania. Fish were monitored and sampled at three stages: low AGD prevalence, high AGD prevalence, and post-freshwater treatment. We analysed gill tissues for gross AGD scores, histopathology scores, *N. perurans* abundance (*18S rRNA* gene expression), and gene expression of inflammation-related transcripts, including angiogenin-1 (*ANG1*), ladderlectin (*LL*), interleukin 8 (*IL8*), glutathione peroxidase 6-like (*GPX6*), and complement C1q tumour necrosis factor-related protein 3-like (*C1QTNF3*). The purpose of this research was to enhance understanding of AGD progression and improve management strategies for gill health in aquaculture. Our findings showed significant correlations between AGD-related gill pathology measures and infection stages. Post-treatment variability highlighted the need for personalized management strategies. *ANG1* and *C1QTNF3* emerged as potential early biomarkers for gill damage in salmon aquaculture.

## 2. Materials and Methods

### 2.1. Fish Husbandry and Sampling Regime

The study focused on sea-farmed Atlantic salmon (*Salmo salar*) experiencing an outbreak of AGD in Tasmania. The fish were cultivated within a semi-commercial open sea-based system utilizing 18 net pens (5 m L × 5 m W × 5 m D), spanning from December 2019 to May 2020. During this period, the mean weekly water temperature, salinity, and dissolved oxygen concentration varied from 13.9 to 16.6 °C, 32.5 to 33.5 ppt, and 6.0 to 9.9 mg/L, respectively, with all parameters measured at a depth of 5 m. Pens were stocked with 270 fish at the start, each weighing 165–170 g, with a survival rate of over 86%. Weekly surveillance for AGD signs was conducted through visual inspection and non-destructive scoring of gill tissue for gross morphology abnormalities. This assessment, carried out by fish health professionals, was based primarily on the proportion of the gill surface occupied by mucus patches, hyperplasia, and other gross morphological changes indicative of AGD progression. The semi-quantitative 6-grade scoring system for AGD ranged from 0 (indicating no observable macroscopic pathology) to 5 (reflecting severe macroscopic pathology), as previously outlined [31]. Each of the eight gill arches (four on the left and four on the right side) underwent separate scoring, resulting in a total of eight AGD scores attributed to each fish. The AGD scores from the eight gill arches were then averaged to generate a single AGD score per fish.

**Figure 1:**
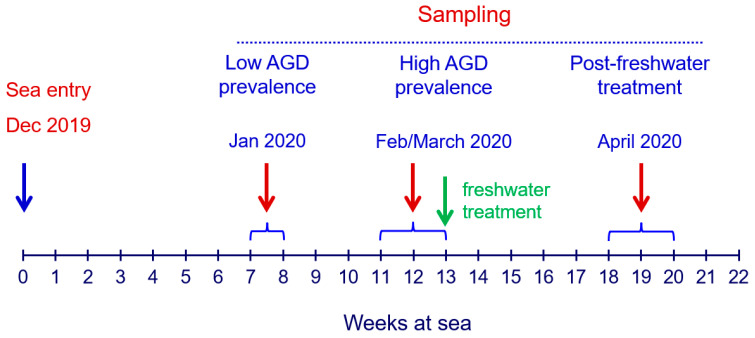
Timeline of the study showing seawater entry, three sampling points and freshwater treatment in farmed Atlantic salmon experiencing an outbreak of AGD in Tasmania (for details see Table S1).

The progression of AGD scores facilitated the identification of different stages within the outbreak, including periods of low and high AGD prevalence, and informed the optimal timing for AGD treatment. A commercial 3-h freshwater bath (salinity < 3 ppt) was administered in a small net pen enclosed with a tarpaulin when average AGD scores exceeded 2. All sampling procedures were conducted by personnel from BioMar Australia. Sampling occurred on three distinct occasions: during the low AGD prevalence stage (at week 7 post-seawater transfer), during the high AGD prevalence stage (weeks 11–13 post-seawater transfer, preceding freshwater treatment), and post-freshwater treatment at the conclusion of the production cycle (weeks 18–20 post-seawater transfer) (Figure 1).

At each sampling point, 4 out of the 18 net pens were selected, and 5 fish were sacrificed from each pen, resulting in 20 fish per sampling and 60 fish in total across the study. The selection of pens was random: 4 of the 18 pens for the first sampling, 4 of the remaining 14 pens for the second sampling, and 4 of the previously used 8 pens for the third sampling, including 2 from the first sampling and 2 from the second sampling (Table S1). The fish from each pen were sampled using a box net, which is the standard approach for subsampling from marine salmon cages. After being caught, the fish were placed in an anaesthetic bath (~20 g of MS-222/150 L) for 5–10 min, followed by macroscopic scoring of gill tissue for AGD manifestation (Table S1). Post-scoring, fish were promptly bled, and the gill arch exhibiting the highest AGD score was excised for subsequent gene expression profiling and histopathological examination, as previously described [9]. Specifically, for gene expression profiling, three transverse sections from the dorsal, medial, and ventral regions of the gill were submerged in RNAlater (Sigma-Aldrich, St. Louis, MO, USA), subjected to an overnight equilibration period at 4 °C, and subsequently stored at −80 °C until RNA extraction. The remaining tissue from the same gill arch was immersed in freshly prepared seawater Davidson’s fixative [45] for 24 h, followed by preservation in 10% neutral buffered formalin before undergoing tissue processing for histopathological examination.

Additionally, the body weights of sampled fish were recorded (Table S1), with averages of 1.1 ± 0.2 kg, 1.4 ± 0.3 kg, and 2.2 ± 0.4 kg observed for the low AGD prevalence, high AGD prevalence, and post-freshwater treatment stages of the outbreak, respectively (*n* = 20, mean ± standard deviation). Following the collection of all gill samples (*n* = 60), they were transferred to the University of Aberdeen (Scotland, UK) for subsequent processing.

### 2.2. Gill Histopathology

Gill tissue was subjected to standard histological procedures, including dehydration in ethanol, equilibration in xylene, and embedding in paraffin wax, following established protocols [46]. Sagittal sections (3 µm) of the gill arch were cut using a microtome and subsequently affixed onto microscope slides. These sections underwent haematoxylin and eosin (H&E) staining to facilitate detailed examination. Utilizing the Olympus dotSlide 2.1 Virtual Slide System (Olympus Corporation, Tokyo, Japan), all stained sections were digitized at a magnification of 40×. To ensure unbiased evaluation, the resultant images were randomized before being subjected to scoring. For this purpose, we employed a semi-quantitative scoring system specifically designed for assessing gill histopathology in sea-farmed Atlantic salmon [16], with minor adaptations from previous work [9].

The gill histopathology scoring system comprised four index criteria and twelve ancillary criteria. Each criterion was assigned a score ranging from 0 (indicating no pathological changes) to 4 (indicating severe changes affecting more than 50% of the gill tissue), with intermediate scores reflecting varying degrees of severity (Table S1). Of particular interest were the index criteria, which included lamellar hyperplasia (LH), lamellar fusion (LF), lamellar oedema (LO), and cellular anomalies (CA), as these features were closely associated with AGD-related lesions [25] (for representative images of gill histopathology see Figure S1). For each fish, the individual scores for LH, LF, LO, and CA were totalled to provide an overall representation of gill histopathology. This approach ensured a robust assessment of the gill tissue condition in relation to AGD. One of the 60 gill samples yielded no histological results due to suboptimal sectioning, which rendered the slide unreadable (Table S1).

### 2.3. RNA Extraction and cDNA Synthesis

Upon thawing, all gill samples (*n =* 60) were trimmed to remove arch tissue while retaining full-length filaments for subsequent processing. Total RNA extraction involved homogenizing ~100 mg of gill filaments (representing the dorsal, medial, and ventral regions of the gill) in TRI Reagent^®^ (Sigma-Aldrich, London, UK), utilizing 3 mm tungsten carbide beads and the TissueLyser II Disruption System (Qiagen GmbH, Hilden, Germany). Following isolation, RNA quantification was performed using spectrophotometry (NanoDrop Technologies, Wilmington, DE, USA), with validation of integrity via electrophoresis (Agilent Technologies, Santa Clara, CA, USA). Subsequently, all RNA samples were subjected to reverse transcription using the QuantiTect RT Kit (Qiagen GmbH, Hilden, Germany) and following the manufacturer’s guidelines. The resulting cDNA samples were diluted to a working concentration of 500 ng/μL with molecular grade water and stored at −20 °C prior to gene expression analysis by qPCR.

### 2.4. Detection of N. perurans and Expression of Host Genes in Gill Tissue

We detected and quantified *N. perurans* in infected gill tissues using targeted amplification of the 18S ribosomal RNA (rRNA) gene, following previously described methods [35,37]. Additionally, we assessed the expression levels of seven salmon genes, including five associated with gill inflammation in Scottish farmed fish [9] and two commonly used as housekeeping genes (Table 1). The expression levels of *N. perurans* and salmon genes were analysed using qPCR.

Briefly, qPCR reactions were performed in 96-well plates, with each reaction containing 7.5 μL of 2× SYBR Green (Agilent Technologies, Cedar Creek, TX, USA), 1.5 μL of forward and reverse primer each (500 nM), 1 μL of molecular grade water, and 5 μL of cDNA (for primer sequence see Table 1). The qPCR was performed using an Mx3005P qPCR System (Agilent Technologies, Santa Clara, CA, USA) with the following cycling parameters: 95 °C for 3 min followed by 40 cycles of 95 °C for 20 s and 64 °C for 20 s (a two-step qPCR). A melting curve was added to the end of every run to confirm the presence of a single PCR product. Expression data were pre-processed and analysed using GenEx Pro Version 5.3.6 (MultiD Analyses AB, Goteborg, Sweden). The cycle threshold (Ct) values of target genes were normalised to the expression of housekeeping genes by ΔCt method. Specifically, the *N. perurans 18S rRNA* gene was normalised to *EF1A*, while the salmon genes associated with inflammation were normalised to *EF1A* and *RPS13*. Relative expression data were log-transformed for statistical analysis. Some qPCR reactions did not reach the quality threshold due to unsatisfactory melting curves and were removed from further analysis (Table S1).

### 2.5. Statistical Analysis

Gill scores are reported as median and interquartile range. Differences in gill scores between fish at the low AGD prevalence, high AGD prevalence, and post-freshwater treatment sampling points were assessed using the Kruskal-Wallis H test, followed by Dunn’s test for subsequent post hoc pairwise comparisons between the groups. The association between gross AGD gill scores and gill histopathology scores, as well as the association between gill scores and the abundance of *N. perurans* as determined by qPCR, was evaluated using Spearman’s rank correlation test.

Gene expression data were assessed for normality using the Shapiro-Wilk test. Due to the non-normal distribution of the data, differences in gene expression across groups were analysed using the Kruskal-Wallis H test. Where necessary, Dunn’s post hoc test was employed for pairwise comparisons. All statistical analyses were conducted using R version 4.2.2 [47]. Statistical significance was established at a threshold of *p* < 0.05.

## 3. Results

### 3.1. Gill Scores

**Figure 2:**
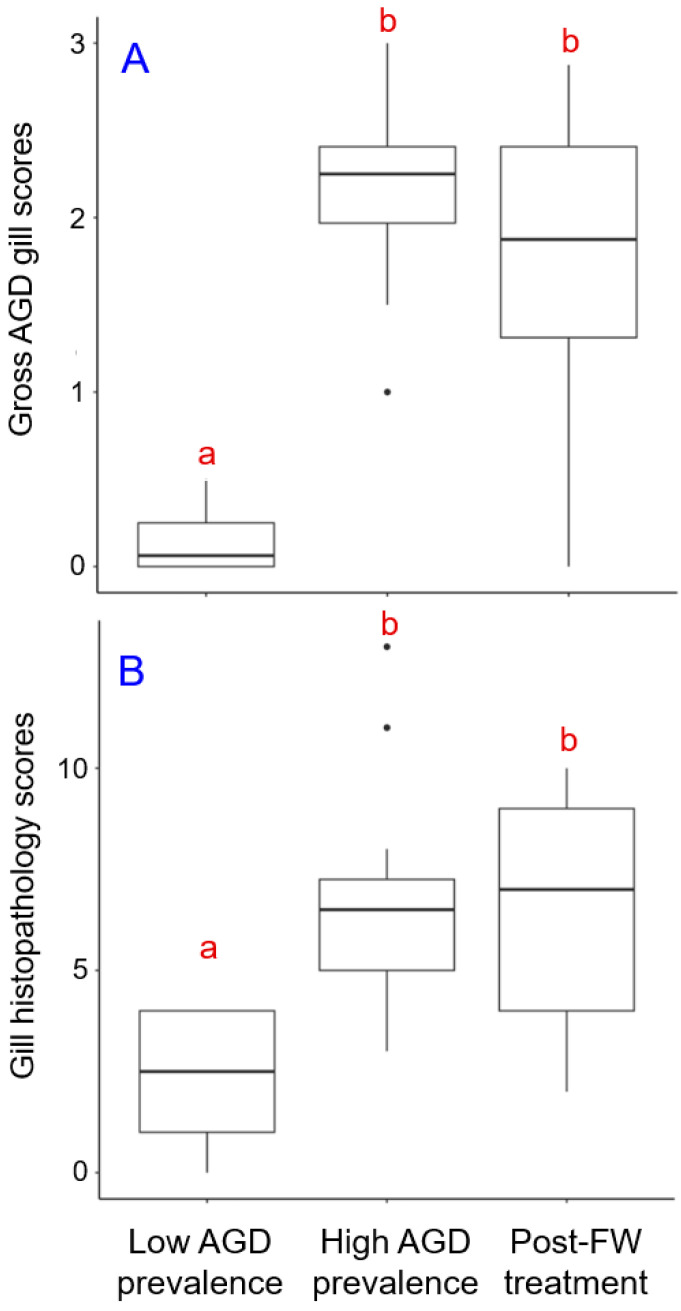
Gross AGD gill scores (**A**) and gill histopathology scores (**B**) in farmed Atlantic salmon at the low AGD prevalence, high AGD prevalence, and post-freshwater (FW) treatment sampling points. Data are presented as boxplots indicating the median, interquartile range (box), 1.5 times the interquartile range (whiskers), and outliers (closed circles). Different letters (a and b) indicate significant differences between the fish groups at *p* < 0.001.

The median gross AGD gill scores of fish sampled during the low AGD prevalence, high AGD prevalence, and post-treatment stages of the outbreak were 0.1 (0.0–0.3), 2.3 (2.0–2.4), and 1.9 (1.3–2.4), respectively (Figure 2A). Statistical analysis revealed significant differences in AGD scores between the fish groups (K-W chi-squared = 36.4, *p* < 0.001). Specifically, fish at both the high AGD prevalence and post-treatment sampling points had significantly higher AGD scores compared to fish at the low AGD prevalence sampling point (low vs. high, *Z* = −5.8, *p* < 0.001; low vs. post-treatment, −*Z* = 4.3, *p* < 0.001). Despite exposure to freshwater treatment (Figure 1), the AGD scores in the high AGD prevalence and post-treatment groups remained indistinguishable (*Z* = 1.5, *p* > 0.05), likely attributed to heterogeneity of fish sampled at the post-treatment time point. Notably, the post-treatment group comprised individuals with gills ranging from healthy (average AGD score of 0) to visibly compromised (average AGD score > 2.5) (Table S1).

The median gill histopathology scores (the sum of index criteria) for fish sampled during the low AGD prevalence, high AGD prevalence, and post-treatment time points were 3.0 ± (1.5–4.0), 6.5 (5.0–7.3), and 7.0 (4.0–9.0), respectively, with a sample size of 19–20 individuals per group (Figure 2B). Differences in gill histopathology scores between the groups were significant (K-W chi-squared = 25.9, *p* < 0.001). Similarly to the AGD scores, the histopathology scores for fish sampled during the high AGD prevalence and post-treatment time points were significantly higher than those in the low AGD prevalence group (low vs. high, *Z* = −4.5, *p* < 0.001; low vs. post-treatment, *Z* = −4.4, *p* < 0.001). Differences in gill histopathology scores between the high AGD prevalence and post-treatments groups were not significant (*Z* = −0.1, *p* > 0.05).

**Figure 3:**
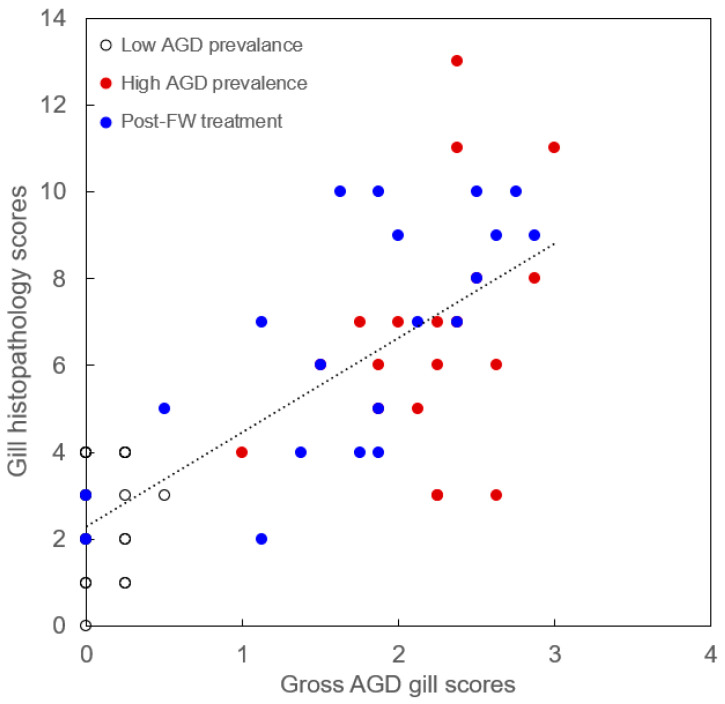
Correlation between gross AGD gill scores and gill histopathology scores in farmed Atlantic salmon at the low AGD prevalence, high AGD prevalence and post-freshwater (FW) treatment sampling points. A linear trendline illustrates a positive association between the two types of gill scores.

The association between gross AGD gill scores and gill histopathology scores was assessed by pooling data from all sampled fish across three time points (*n* = 59). Analysis revealed a strong and significant positive correlation between AGD gill scores and gill histopathology scores (Spearman’s *p* = 0.76, *p* < 0.001), indicating that fish with higher AGD gill scores tended to exhibit more severe gill histopathology (Figure 3).

### 3.2. Abundance of N. perurans in Gill Tissue

**Figure 4:**
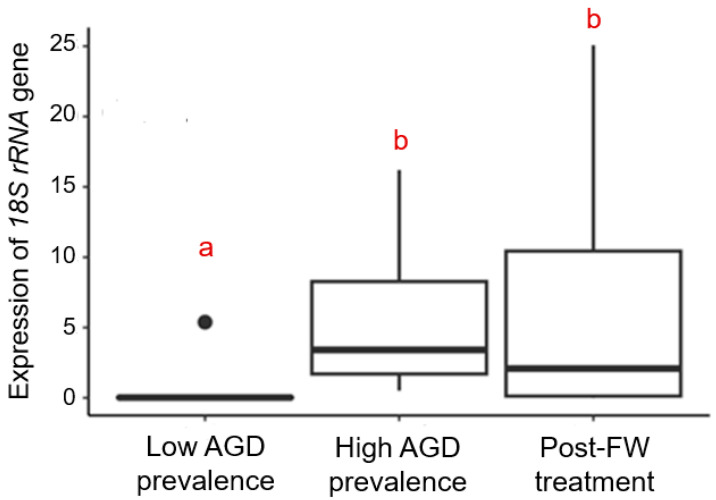
Abundance of *N. perurans* in the gill tissue of Atlantic salmon at the low AGD prevalence, high AGD prevalence, and post-freshwater (FW) treatment sampling points, quantified as the expression levels of *N. perurans 18S rRNA* gene (relative units) by qPCR. Data are presented as boxplots indicating the median, interquartile range (box), 1.5 times the interquartile range (whiskers), and outliers (closed circles). Different letters (a and b) indicate significant differences between the fish groups at *p* ≤ 0.001.

The abundance of *N. perurans* in the gill tissue was quantified by measuring the expression levels of the *N. perurans 18S rRNA* gene, normalised to the expression of the housekeeping gene *EF1A* (Table 1). Fish sampled at three distinct time points exhibited significantly different levels of the *18S rRNA* transcript (K-W chi-squared = 20.5, *p* < 0.001). Specifically, expression levels of the *18S rRNA* transcript were nearly undetectable at the low AGD prevalence sampling point but increased significantly during the high AGD prevalence and post-treatment time points (low vs. high, *Z* = −4.2, *p* < 0.001; low vs. post-treatment, *Z* = −3.3, *p* = 0.001). However, the differences between the high AGD prevalence and post-treatment sampling points were not statistically significant (*Z* = 0.8, *p* > 0.05) (Figure 4).

**Figure 5:**
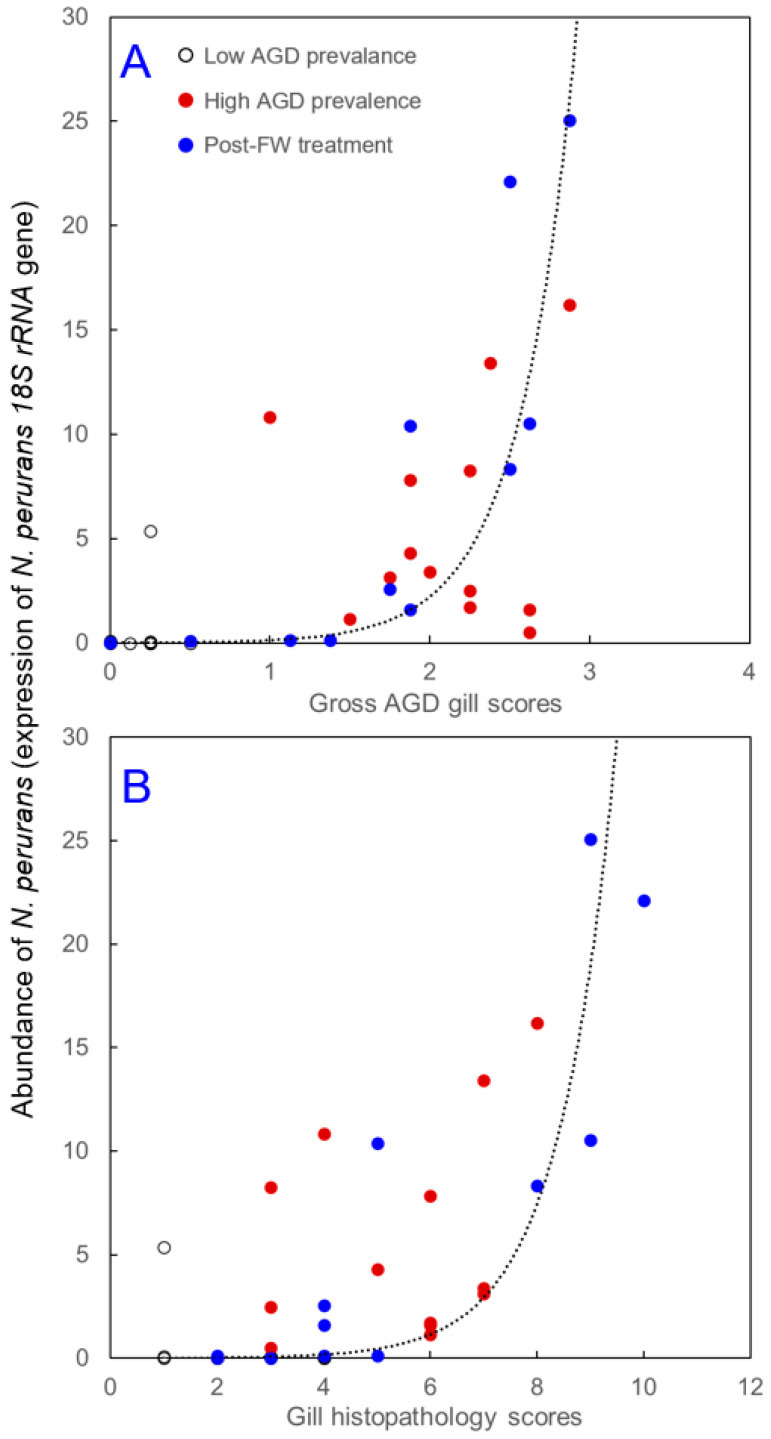
Correlation between the abundance of *N. perurans* in gill tissue and gross AGD gill scores (**A**), and gill histopathology scores (**B**) in farmed Atlantic salmon at the low AGD prevalence, high AGD prevalence, and post-freshwater (FW) treatment sampling points. An exponential trendline illustrates a monotonic increase in *N. perurans* abundance corresponding to rising gill scores.

Pooling data from all sampled fish across three time points revealed significant correlations between the abundance of *N. perurans* in gill tissue and gill scores. Fish with higher gross AGD gill scores exhibited a higher abundance of *N. perurans* (Spearman’s *ρ* = 0.80, *p* < 0.001, *n* = 41) (Figure 5A). A similar association was also found for gill histopathology scores (Spearman’s *ρ* = 0.66, *p* < 0.001, *n* = 40) (Figure 5B).

### 3.3. Expression of Salmon Genes

**Figure 6:**
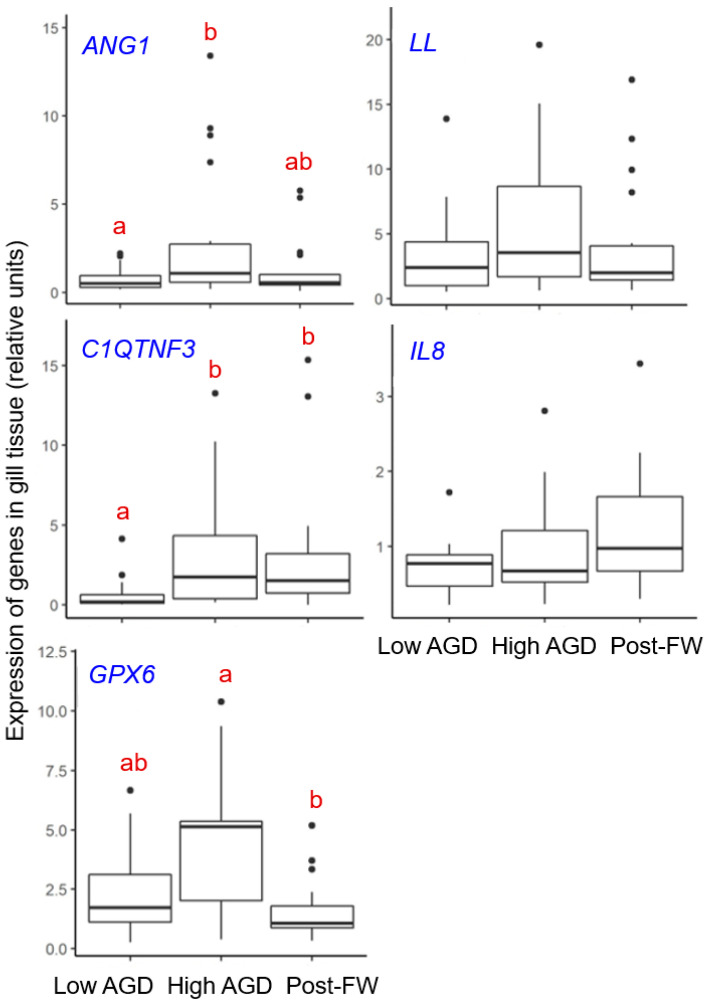
Gene expression levels of five inflammation-related genes (*ANG1*, *C1QTNF3*, *GPX6*, *LL*, *IL8*) in the gill tissue of farmed Atlantic salmon at the low AGD prevalence, high AGD prevalence, and post-freshwater (FW) treatment sampling points. Data are presented as boxplots indicating the median, interquartile range (box), 1.5 times the interquartile range (whiskers), and outliers (closed circles). The genes in the left panel (*ANG1*, *C1QTNF3* and *GPX6*) showed different expression levels across the stages, with distinct letters (a and b) denoting statistically significant differences among the groups. The genes in the right panel (*LL* and *IL8*) showed no significant differences in expression between the groups.

Three of the five inflammation-related genes showed significant differences in the expression levels between fish sampled at different stages of *N. perurans* infection. These genes included *ANG1* (K-W chi-squared = 7.0, *p* = 0.031, *n* = 55), *C1QTNF3* (K-W chi-squared = 12.0, *p* = 0.003, *n* = 51) and *GPX6* (K-W chi-squared = 8.9, *p* = 0.011, *n* = 52) (Figure 6). In contrast, the two remaining genes (*LL* and *IL8*) showed no such differences (K-W chi-squared < 4.2, *p* > 0.05, *n* = 53–55).

The most prominent feature of the observed gene expression changes was an increase in gene expression from relatively lower levels during the low AGD prevalence stage to relatively higher levels during the high AGD prevalence stage of *N. perurans* outbreak. For genes *ANG1* and *C1QTNF3*, the increase was significant (*ANG1*, low vs. high, *Z* = −2.6, *p* = 0.016; *C1QTNF3*, low vs. high, *Z* = −3.1, *p* = 0.003), while for *GPX6*, the increase did not reach significance (low vs. high, *Z* = −1.9, *p* > 0.05). Post-treatment, the transcript levels were either indistinguishable from the low and high AGD prevalence levels (ANG1; low vs. post-treatment, *Z* = −0.7, *p* > 0.05; high vs. post-treatment, *Z* = 1.8, *p* > 0.05), remained elevated (*C1QTNF3*; low vs. post-treatment, *Z* = −2.9, *p* = 0.005; high vs. post-treatment, *Z* = 0.2, *p* > 0.05), or dropped down to the low AGD prevalence levels (*GPX6*; low vs. post-treatment, *Z* = 1.2, *p* > 0.05; high vs. post-treatment, *Z* = 3.0, *p* = 0.005) (Figure 6).

## 4. Discussion

Atlantic salmon aquaculture faces increasing challenges from climatic shifts and disease outbreaks, particularly those affecting gill health [5,6,7,48]. As the industry intensifies, it is crucial to enhance measures for monitoring, controlling, and maintaining fish health. Diseases impacting the gills compromise physiological functions, leading to significant economic losses from poor feed conversion, reduced growth rates, treatment costs, and increased mortality [21,24]. Handling and crowding exacerbate gill pathologies, highlighting the need for regular, non-destructive monitoring of sea-farmed fish [49,50]. AGD has become a significant global concern, necessitating effective surveillance and response strategies amid ongoing environmental changes [18,19,51]. This study rigorously assesses gill tissue from sea-farmed Atlantic salmon during an AGD outbreak in Tasmania, addressing knowledge gaps and identifying gene expression patterns associated with AGD-related pathology.

In this study, fish were sampled at three stages of *N. perurans* outbreak: low AGD prevalence, high AGD prevalence, and post-freshwater treatment (Figure 1). Evaluations included gross AGD scores, histopathological scores, *N. perurans* abundance by qPCR, and inflammation-related gene expression. While some of these metrics are established for monitoring AGD [16,31,32,33,34,35,36,37], they are rarely applied concurrently. This study addresses this gap and also pioneers gene expression analysis to establish a robust panel of biomarkers for gill health, enhancing the understanding and management of gill pathologies in aquaculture [9].

When different measures of AGD-related gill pathologies were applied concurrently to fish experiencing an outbreak of AGD, they exhibited a remarkably similar pattern of temporal changes. Specifically, the gross AGD gill scores, gill histopathology scores, and the abundance of *N. perurans* were relatively low in fish sampled during the low AGD prevalence stage of the outbreak, then significantly increased during the high AGD prevalence stage (Figure 2 and Figure 4). Interestingly, while the freshwater treatment seemed to halt the further increase in these measured parameters, it did not return them to the low AGD prevalence levels. Instead, the freshwater bath led to increased variability in both gill scores and *N. perurans* abundance among individual fish around the group median, as evidenced by the expanded interquartile ranges and the broader spread of minimum and maximum values in the boxplots (Figure 2 and Figure 4). Across all three stages of *N. perurans* outbreak, the gross AGD gill scores and gill histopathology scores were highly and positively correlated with each other (Figure 3). This finding contrasts with our earlier results on the limited association between gross gill morphology and gill histopathology in Atlantic salmon farmed in Scotland [9]. The gills of Scottish-farmed salmon were macroscopically scored for multifactorial gill pathologies, whereas the fish in the current study were specifically scored for AGD, explaining the observed differences. Previously, gross gill scores have been challenging to interpret when non-AGD pathologies such as CGD are present [15,52]. Finally, our study demonstrated that both macroscopic and microscopic gill scores correlate well with the abundance of *N. perurans* (Figure 5), consistent with previous research [32,34].

Post-freshwater treatment, fish exhibited large variability in their responses to the freshwater bath, ranging from individuals with healthy gills to those with significantly compromised gills (Figure 2 and Figure 4). Specifically, the gross AGD gill scores, gill histopathology scores, and *N. perurans* abundance varied from 0 to 2.9, 2 to 10, and 0 to 25.1, respectively (Table S1). This variability can be attributed to several factors that not only influence the health outcomes of individual fish but also their propensity for reinfection following treatment [41]. Firstly, subtle genetic differences among the fish may lead to varied immune responses to *N. perurans*, affecting their susceptibility to AGD and recovery post-treatment [53]. Secondly, environmental conditions within the aquaculture system, such as water quality, temperature, and oxygen levels, can differ slightly from one area to another. These microenvironmental variations can significantly impact disease dynamics and the effectiveness of the freshwater treatment across different groups of fish [54]. Thirdly, previous health history may also play crucial roles. Fish that have encountered previous stressors or health challenges may have compromised gill structures, making them more susceptible to severe AGD manifestations and less responsive to treatment [55]. Moreover, the stage of infection at the time of treatment can greatly influence outcomes. Fish treated at an earlier stage of infection might display minimal pathological changes and recover more rapidly, whereas those treated at more advanced stages might not recover as effectively, leading to higher variability in gill scores and pathogen abundance [38]. Understanding this high degree of variability is crucial for optimizing treatment protocols and improving overall fish health management in aquaculture settings.

While scoring gill tissue for macroscopic and microscopic features serves as a reliable monitoring tool for AGD-related pathologies, such gill scores above 0 indicate tissue that is already visibly damaged [16,31,33]. Recognizing the limitations of these traditional methods, recent studies have demonstrated that changes in gene expression can precede visible tissue damage [9,56,57]. This suggests that monitoring gene expression in gill tissue could serve as an effective early warning system, potentially allowing for pre-emptive interventions. Our earlier work identified a panel of potential biomarkers of gill health developed for Scottish-farmed Atlantic salmon experiencing multifactorial gill pathologies [9]. In the current study, we evaluated five of these gill inflammation-related transcripts (Table 1). Three of them (*ANG1*, *C1QTNF3* and *GPX6*) showed differences across various stages of *N. perurans* outbreak, but only two transcripts (*ANG1* and *C1QTNF3*) were differentially expressed between the low and high AGD prevalence stages (Figure 6), which correspond to the most significant changes in gill pathology and pathogen abundance (Figure 2 and Figure 4). The higher gene expression of *ANG1* during the high AGD prevalence stage (Figure 6) is consistent with higher protein levels of angigenin-1 in the gills of Atlantic salmon following four successive infections with *N. perurans* [58]. In fish, angiogenin-related proteins are involved in tissue regeneration and repair, immune responses, and possibly in the development of blood vessels [59]. Given its functions in other vertebrates, *ANG1* might also contribute to physiological processes like wound healing and response to infection in fish [9,60]. Similarly to *ANG1*, the expression of *C1QTNF3* increased from relatively low levels during the low AGD prevalence stage to significantly higher levels during the high AGD prevalence stage (Figure 6). The *C1QTNF3* gene encodes a protein that is crucial to the complement system, a fundamental part of the innate immune response [61]. This protein primarily functions to inhibit the complement system, preventing its overactivation and thus reducing excessive inflammation and subsequent tissue damage. Notable changes in *C1QTNF3* gene expression have been demonstrated in the gill epithelia of tilapia following salinity challenges [62], and in the fins of Atlantic salmon infested with sea lice [63]. The consistent upregulation of *ANG1* and *C1QTNF3* transcripts in the gill tissues of Scottish-farmed Atlantic salmon with multifactorial gill pathologies [9] and Tasmanian Atlantic salmon affected by AGD (this study) underscores their potential as biomarkers for early detection of gill damage in salmon aquaculture worldwide.

## 5. Study Limitations

One limitation of our study is the time gap between the freshwater treatment and the sampling, which was 5–7 weeks. This delay could have influenced the results, as the potential for reinfection during this period might have contributed to the variability in the gross AGD gill scores, gill histopathology scores, and the abundance of *N. perurans*. While we acknowledged the possibility of reinfection, the impact of this time gap on distinguishing between acute and chronic infections was not fully explored. Future studies should aim to sample more frequently post-treatment to better understand the immediate and long-term effects of freshwater treatments on AGD.

Another limitation is the absence of a control group that did not receive freshwater treatment. Without this control group, it is difficult to assert definitively that the observed stabilization in measured parameters was due to the treatment itself. The values might have stabilized even without the treatment. Including a control group in future studies would help in drawing more concrete conclusions about the efficacy of freshwater treatments.

Additionally, our study did not include initial control values before the onset of low AGD prevalence. This absence makes it challenging to compare the initial values with the low AGD values and to use these parameters as reliable indicators for early detection. Future studies should ensure the inclusion of baseline measurements before the onset of AGD to establish a clear reference point. This would enable a more accurate assessment of disease progression and the effectiveness of early detection of measures for AGD.

## 6. Conclusions

Our critical evaluation of AGD progression through a combined approach of gross morphological scoring, histopathological assessments, and molecular diagnostics, including qPCR and gene expression profiling, not only enhances our understanding of the disease dynamics but also facilitates the development of targeted intervention strategies. Specifically, the identification of potential biomarkers of gill health such as *ANG1* and *C1QTNF3* offers promising avenues for the early detection and management of gill pathologies, potentially enabling pre-emptive actions before the onset of critical disease stages.

Moreover, the variability in the responses of individual fish to freshwater treatment underscores the need for personalized management strategies that consider genetic, environmental, and health status factors. Integrating comprehensive monitoring systems alongside traditional and emerging treatment methodologies is imperative to improve resilience against AGD. Future research should focus on optimizing these strategies to mitigate the impact of AGD and ensure the sustainability of salmon farming under changing climatic conditions. The insights gained from this study contribute significantly to the body of knowledge required to safeguard the health of farmed Atlantic salmon and enhance the economic viability of the aquaculture industry.

## Figures and Tables

**Table 1:** List of genes and primers used for qPCR in gill tissue of Atlantic salmon.

Gene Symbol ^1^	Gene Name	Species	NCBI Accession Number	Ensembl Accession Number	Primers 5′-3′ (Forward and Reverse) ^2^
*18S rRNA* gene	18S ribosomal RNA gene	*N. perurans*	EF216905	n/a	F:GTTCTTTCGGGAGCT R:GAACTATCGCCGGCA
*ANG1*	Angiogenin-1	*S. salar*	XM_014199172	ENSSSAT00000023457	F:ACTGTGGCAGATATTTGGGGAAGA R:GTCACCCTGGACACCTGTGG
*LL*	Ladderlectin	*S. salar*	XM_014125761	ENSSSAT00000132935	F:GATCTACGTGCCGCAAAGGC R:TTTGGTCCAACCTCCGGGAC
*IL8*	Interleukin 8	*S. salar*	NM_001140710	ENSSSAT00000013831	F:GAATGTCAGCCAGCCTTGT R:TCCAGACAAATCTCCTGACCG
*GPX6*	Glutathione peroxidase 6-like	*S. salar*	XM_014155258	ENSSSAT00000067910	F:TAGCATGCAGGGTTACACAATGG R:GAGCACCTTGCCCCTGTAGT
*C1QTNF3*	Complement C1q tumour necrosis factor-related protein 3-like	*S. salar*	XM_014134640	ENSSSAT00000142506	F:AGACGATGCTTCCTCTCCAGAT R:ACACCCACAGAGTTGCGTGA
*EF1* *A*	Elongation factor 1-alpha	*S. salar*	XM_014177562	ENSSSAT00000157959	F:CAAGGATATCCGTCGTGGCA R:ACAGCGAAACGACCAAGAGG
*RPS13*	Ribosomal protein S13	*S. salar*	BT059859	ENSSSAT00000078374	F:CCCTCTCAGATCGGTGTGATCC R:TCCTGTCCTTTCTGTTCCTCTCC

^1^ *ANG1*, *LL*, *IL8*, *GPX6* and *C1QTNF3* were assayed due to their association with gill inflammation [9], *EF1A* and *RPS13* served as housekeeping genes. ^2^ Primers for *N. perurans 18S rRNA* gene were previously published [35], primers for salmon genes were specifically designed for this study.

## Data Availability

All data are presented in Supplementary Materials, Table S1.

## Supplementary Materials

The following supporting information can be downloaded at: https://www.mdpi.com/article/10.3390/ani14162356/s1, Table S1: Sampling details, gill scores and gene expression data for sea-farmed Atlantic salmon (*n =* 60) experiencing an outbreak of amoebic gill disease (AGD) in Tasmania. Figure S1: Representative images of gill histopathology in farmed Tasmanian Atlantic salmon at the high AGD prevalence sampling point.
