# Supplementary material for: Dynamics of Gill Responses to a Natural Infection with Neoparamoeba perurans in Farmed Tasmanian Atlantic Salmon
Source: Animals (Basel). 2024 Aug 15;14(16):2356. doi: 10.3390/ani14162356 (PMC11350870; doi:10.3390/ani14162356)
Supplement: Supplementary file 1 [file animals-14-02356-s001.zip › animals-3086777- supplementary/Figure S1.pdf]

Figure S1. Representative images of gill histopathology in farmed Tasmanian Atlantic salmon at the high AGD prevalence sampling point (Feb/March 2020). The images highlight (**A**) lamellar hyperplasia (LH) and lamellar fusion (LF), (B) lamellar oedema (LO), and (**C**) cellular anomalies (CA). The scale bar represents 200 μm.

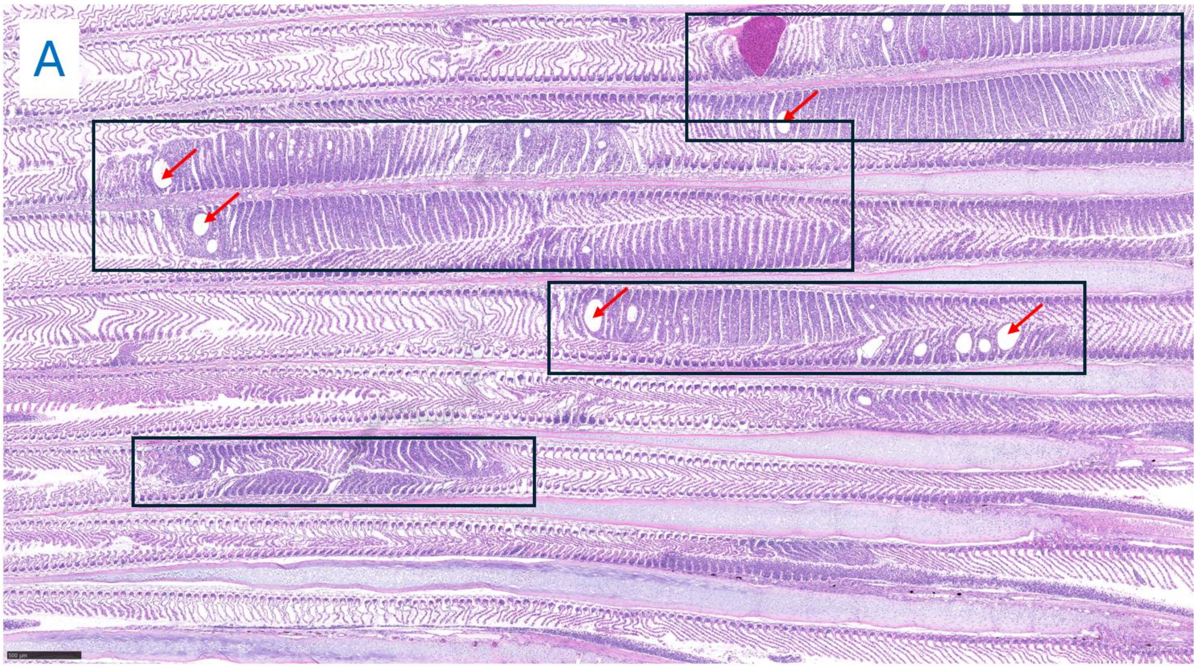

Image A: Multifocal, severe lamellar hyperplasia (LH) and lamellar fusion (LF) (indicated by black rectangles) occurring simultaneously and affecting several filaments. Note the formation of pseudocysts (indicated by arrows), a common feature in AGD-affected gills.

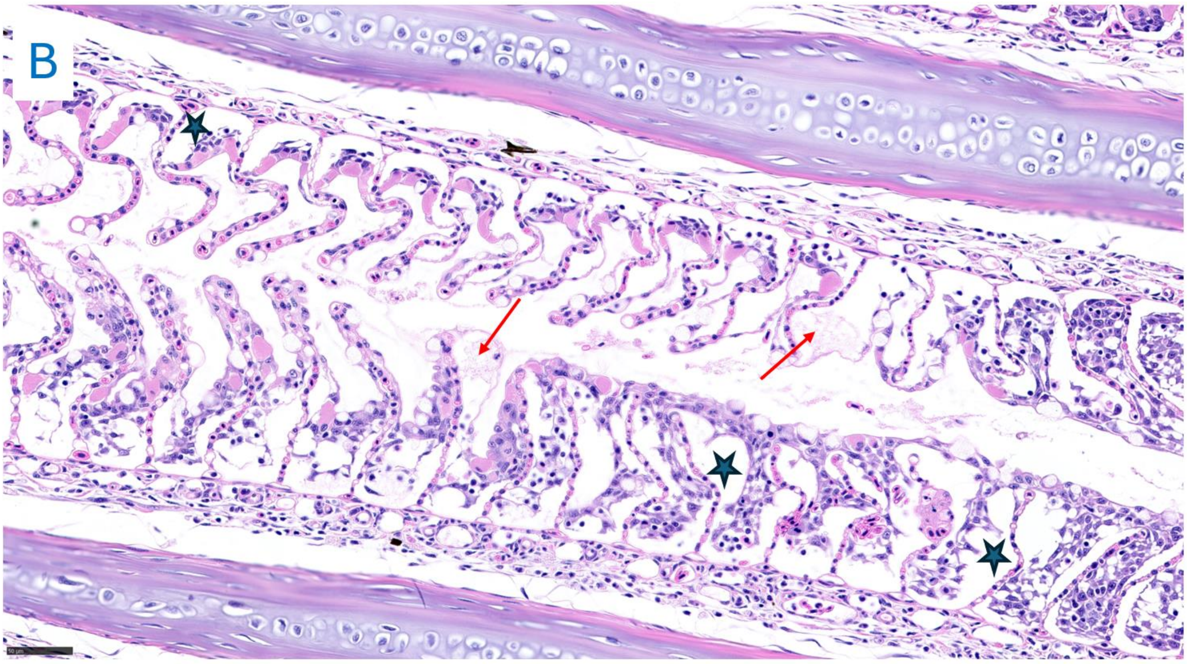

Image B: Lamellar oedema (LO) (indicated by red arrows) contrasted with artefactual epithelial capillary separation (indicated by black stars). Note the mild hyperplasia of the lamellar epithelium and subepithelial inflammation affecting the lower row of lamellae.

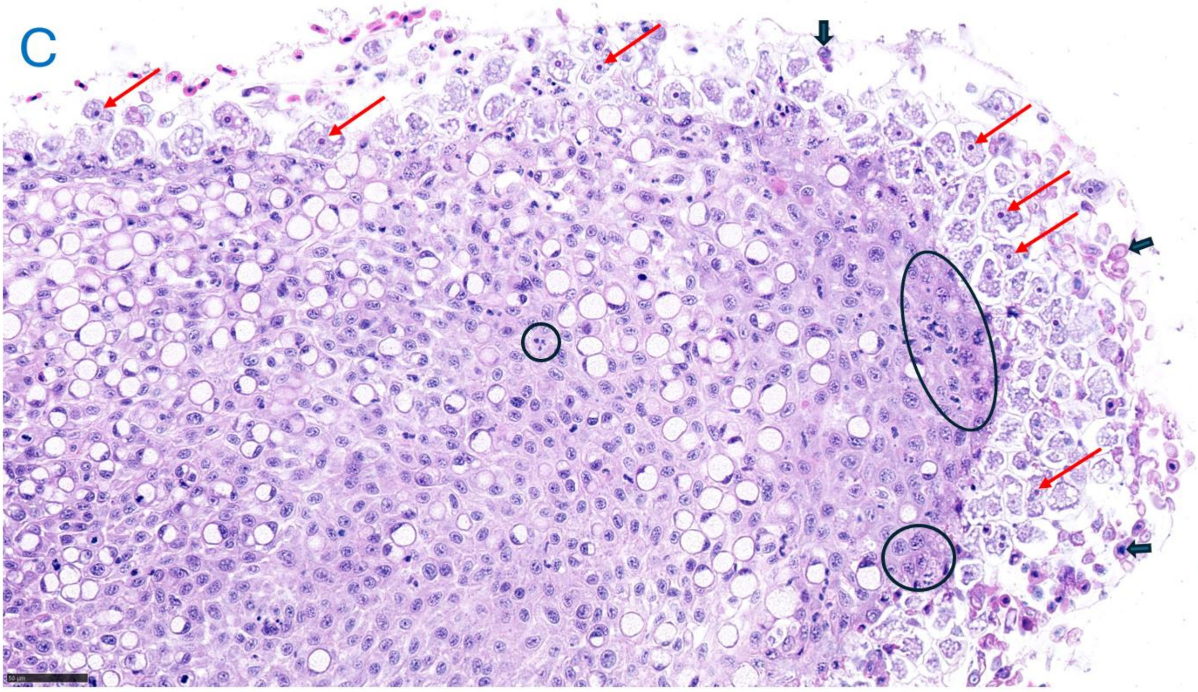

Image C: Cellular anomalies (CA) featuring degenerated and necrotic cells (encircled) and sloughed cells (indicated by black arrows). Note the abundant presence of amoebas (indicated by red arrows).
